# Robot-Assisted Partial Cystectomy Using the “Double Bipolar Method”

**DOI:** 10.7759/cureus.61610

**Published:** 2024-06-03

**Authors:** Shieru Hamasaki, Go Kaneko, Akira Yabuno, Yu Miyama, Shinnosuke Hiruta, Masayuki Hagiwara, Suguru Shirotake, Masanori Yasuda, Masafumi Oyama

**Affiliations:** 1 Uro-Oncology, Saitama Medical University International Medical Center, Hidaka, JPN; 2 Gynecologic Oncology, Saitama Medical University International Medical Center, Hidaka, JPN; 3 Pathology, Saitama Medical University International Medical Center, Hidaka, JPN

**Keywords:** urachal remnant, adult urachal cyst, double bipolar method, partial cystectomy, robot-assisted surgery, da vinci robot, robot, robot assisted surgery

## Abstract

The “double bipolar method” (DBM) in robotic surgery has been widely used in Japanese general surgery and gynecology; however, it is not commonly used in the field of urology. A 55-year-old female was diagnosed with stage IA endometrial cancer. A 2-cm cystic lesion was incidentally observed at the dome of the bladder on magnetic resonance imaging. A simultaneous robot-assisted total hysterectomy and partial cystectomy using the da Vinci Xi system was planned. The gynecological procedure was first performed with the DBM, and the DBM was also used in the partial cystectomy without additional instruments to reduce surgical costs. Maryland bipolar forceps was used to excise the peritoneum, fat, and bladder wall without bleeding, enabling delicate and precise resection using the forceps’ tips. Robot-assisted partial cystectomy using the DBM was feasible. When performing combined surgeries with other departments, if the DBM is already being utilized, it is worthwhile to attempt to decrease surgical cost.

## Introduction

Robot-assisted surgery is spreading across all surgical specialties as insurance coverage expands and new robots become available in Japan. As with laparotomy and laparoscopic surgery, the choice of instruments, energy sources, and technique in robotic surgery varies among institutions, specialties, and surgeons.

The “double bipolar method” (DBM) was first proposed by Uyama et al. [1]. In the procedure, Maryland bipolar forceps and other bipolar forceps are usually controlled by the surgeon’s dominant and non-dominant hands, respectively. Cutting and coagulation vary depending on how the tissue is grasped with the Maryland bipolar forceps. When used with a thick bite, the Maryland bipolar forceps can coagulate the grasped tissue, whereas when used with a thin bite, the tissue can be cut using a spark [2]. This method is mainly utilized in Japanese general surgery and gynecology [3,4]. However, in the Japanese urological field, it is common to use monopolar scissors in one hand and bipolar forceps in the other. The use of the DBM is relatively uncommon. Herein, we present a case of robot-assisted partial cystectomy using the DBM for a cystic lesion located at the bladder dome, which was performed at the same time as robot-assisted hysterectomy and bilateral salpingo-oophorectomy for endometrial cancer.

## Case presentation

**Figure 1:**
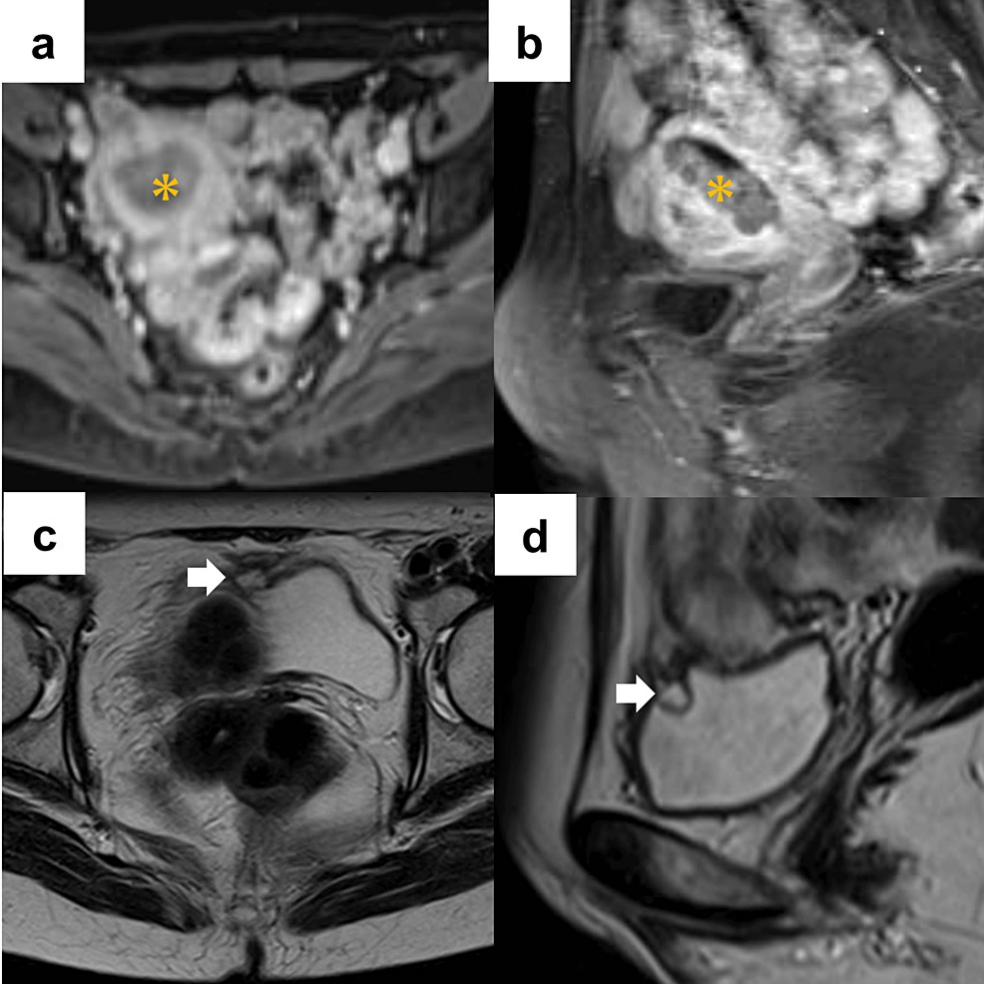
Magnetic resonance imaging of endometrial cancer and bladder cystic lesion a and b: Gadolinium-enhanced magnetic resonance imaging revealed endometrial cancer in stage ⅠA, according to the International Federation of Gynecology and Obstetrics (FIGO) staging classification (*) (a: axial section and b: coronal section). c and d: Magnetic resonance imaging (T2-weighted) showed a 2-cm cystic lesion at the bladder dome (arrow) (c: axial section and d: coronal section).

**Figure 2:**
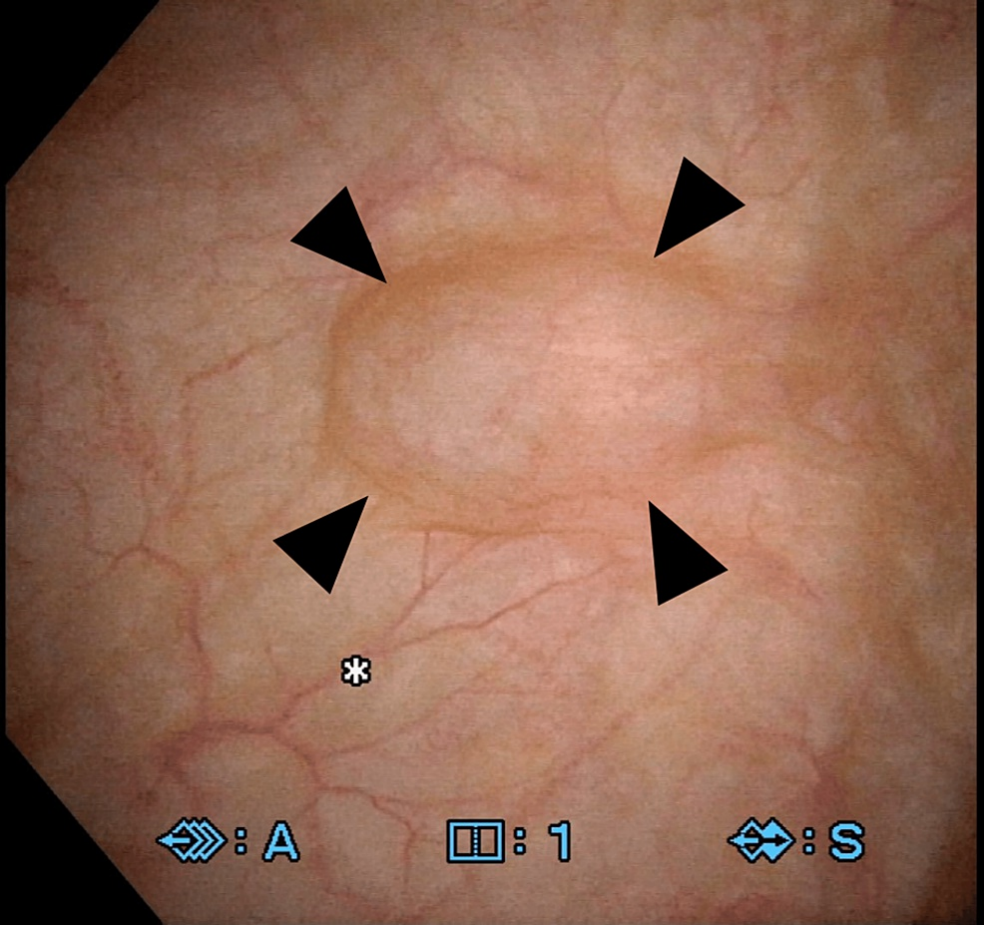
Cystoscopic finding Cystoscopy revealed a protruding lesion covering normal mucosa at the bladder dome (arrowhead).

A 55-year-old female visited a neighboring hospital with complaints of genital bleeding. Endometrial cancer (stage IA, according to the International Federation of Gynecology and Obstetrics staging classification) was suspected on magnetic resonance imaging (Figures 1a, 1b). She was referred to the gynecology department at our hospital for further evaluation. Grade 1 endometrial adenocarcinoma was diagnosed by endometrial biopsy. On re-evaluation with magnetic resonance imaging, a 2-cm cystic lesion was incidentally observed at the bladder dome (Figures 1c, 1d). Cystoscopy revealed a protruding lesion; however, the mucosal surface appeared normal (Figure 2). Based on these findings, urachal cyst was suspected. Simultaneous robot-assisted total hysterectomy, bilateral salpingo-oophorectomy, and partial cystectomy were planned using da Vinci Xi (Intuitive Surgical Inc., Sunnyvale, CA, USA).

**Figure 3:**
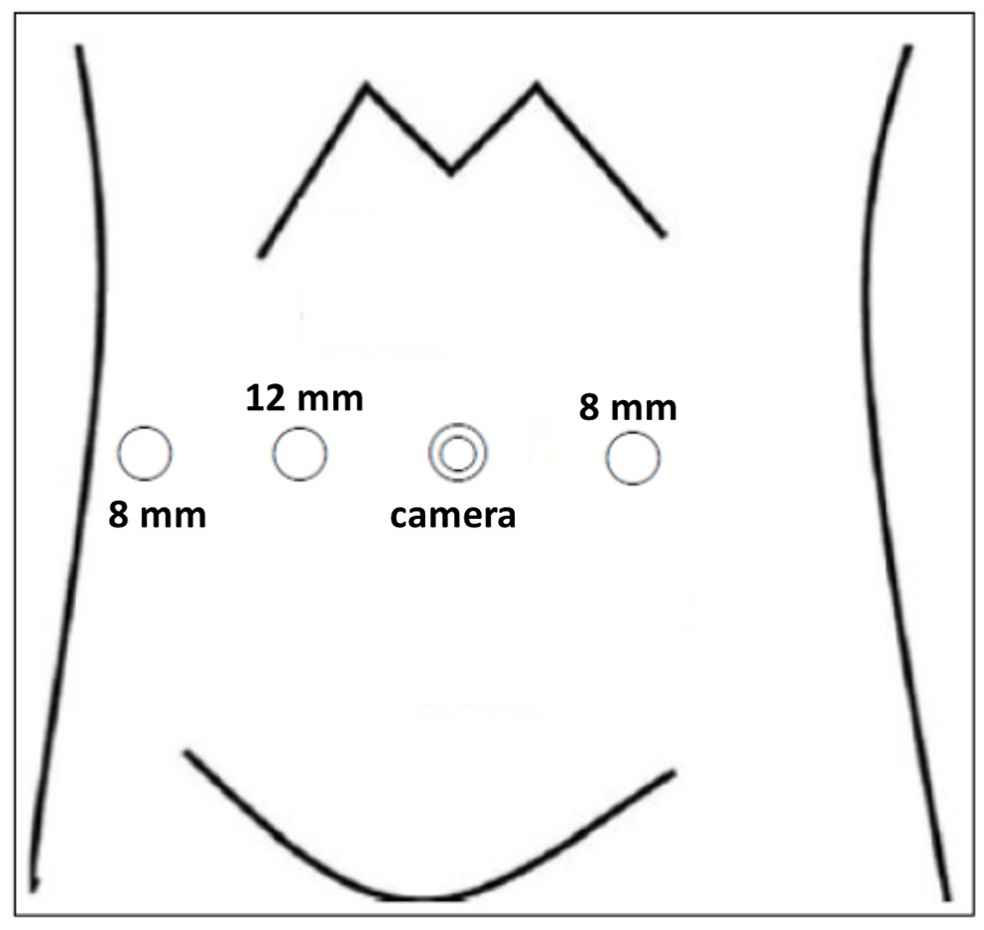
Trocar position All trocars were inserted at the level of the umbilicus with 8 cm between the ports.

**Figure 4:**
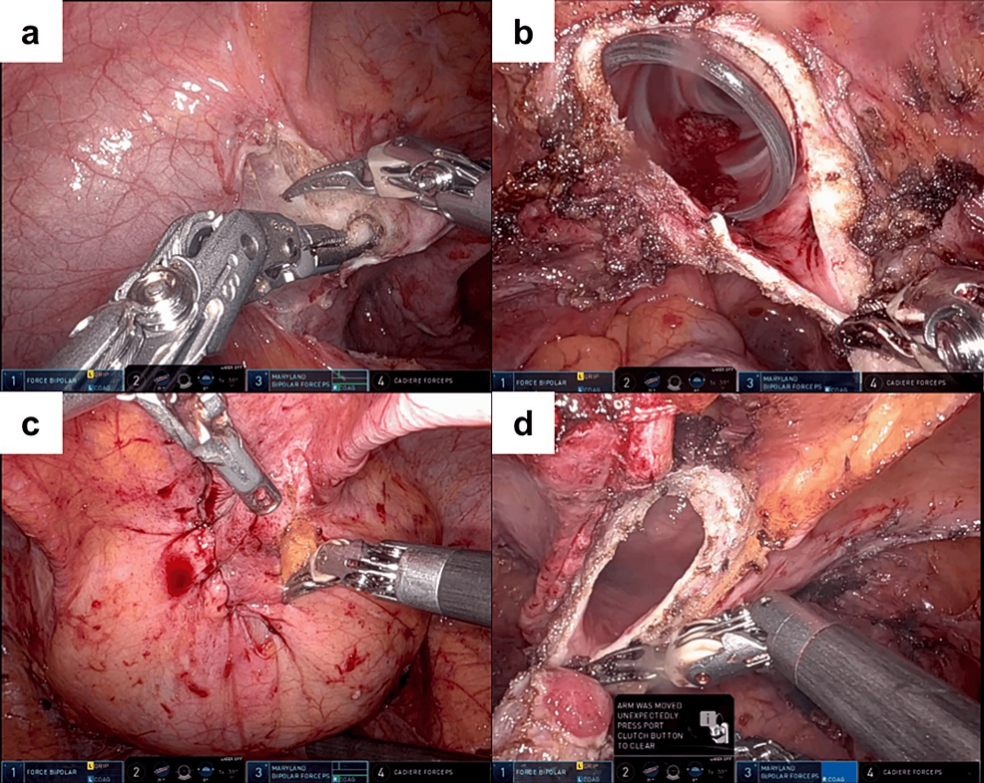
Intraoperative findings Resection using the “double bipolar method” was performed on a) the pelvic peritoneum, b) the vaginal wall, c) the peritoneum covering the bladder, and d) the bladder wall.

**Figure 5:**
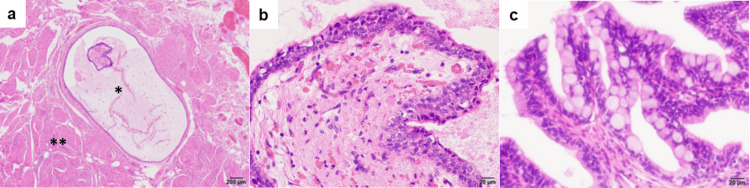
Microscopic findings of the resected specimen a: Hematoxylin and eosin staining, (*: urachal remnant, **: bladder muscle). Scale bar in the lower right corner indicates 200 μm. b and c: The urachal remnant was composed of: b) urothelium-like cells and c) mucus-producing columnar epithelium. Scale bar in the lower right corner indicates 20 μm.

Under general anesthesia, the patient was placed in the 20° Trendelenburg position, and a camera port was inserted at the umbilicus. After establishing pneumoperitoneum, three trocars were inserted at the level of the umbilicus (Figure 3). The distance between the ports was 8 cm. A 12-mm diameter camera, Maryland bipolar forceps, Force bipolar forceps, Cadiere forceps, and Mega suture cut needle driver were used. First, total hysterectomy and bilateral salpingo-oophorectomy were performed in the usual manner using the DBM with Maryland bipolar forceps and Force bipolar forceps (Figures 4a, 4b). Maryland bipolar forceps were connected to the Valleylab FT 10 Energy Platform (Medtronic) in “MACRO mode” at 60 watts. After removal of the resected specimen, robot-assisted partial cystectomy was performed without changing the instruments. A cystoscope was inserted, and the bladder was distended with 200 cc of air. The resection boundaries of the bladder wall were determined, and the resection was performed using the DBM. Maryland bipolar forceps was used to excise the peritoneum (Figure 4c), fat, and bladder wall (Figure 4d) without bleeding, allowing delicate and precise resection using the tips of the forceps. Precise resection could not be achieved if adequate tension was not applied to the excision site or if charred tissue adhered to the tips of the forceps. The bladder defect was closed with a 3-0 spiral polydioxanone barbed suture (STRATAFIXR; Ethicon, Somerville, NJ). The postoperative course was uneventful. A cystogram was performed on the fifth postoperative day to confirm the absence of urine leakage. The urethral catheter was then removed. Once it was confirmed that the patient was urinating normally, they were discharged from the hospital on the sixth postoperative day. The histopathological diagnosis of the partially resected bladder and the uterus was a urachal remnant (Figure 5) and endometrioid carcinoma (Grade 3, pT1a) with a slight observation of lymphatic invasion, respectively.

## Discussion

Herein, we described our experience with robot-assisted partial cystectomy using the DBM. Cutting the bladder wall with Maryland bipolar forceps was a novel procedure for our team. Although excision with Maryland bipolar forceps was time-consuming, it allowed for precise and accurate resection.

As described above, the DBM is widely used in Japanese general surgery and gynecology [1-6]. Its efficacy in robot-assisted gastrectomy has been reported [1,2,6], and the technique is often used due to its excellent surgical outcomes and lower complication rates. The use of the DBM has been reported not only in gastrectomy but also in other surgical procedures such as robotic total mesorectal excision [3]. The console times in cases using the DBM were significantly shorter, and other surgical outcomes, such as estimated blood loss and complication rate, were comparable compared with cases using the conventional “single bipolar method”. The usefulness of the technique has also been reported in more challenging gynecological procedures. Kanno et al. reported the technical feasibility and preservation of postoperative bladder and rectal function in nerve plane-sparing eradication of deep endometriosis [4]. The reported usefulness of the DBM in abdominal and pelvic surgery suggests that it may also be useful in urological surgery.

Robot-assisted partial cystectomy has been reported for urachal disease, using monopolar scissors to resect the bladder wall [7-10]. To the best of our knowledge, robot-assisted partial cystectomy using the DBM has not been reported. Excision of the bladder wall using Maryland bipolar forceps was easily performed, and precise resection was achieved without bleeding. This case represents our inaugural experience with the DBM. Consequently, it required time before the optimal tissue tension for bipolar resection was identified. However, by applying the appropriate tension to the tissue and resecting with the tip of the forceps, we were able to resect the bladder wall in a relatively expeditious manner. As experience is accumulated, it is anticipated that partial bladder resection will be possible at the same time as when using monopolar scissors. The DBM may be useful for other urologic procedures; however, it is our contention that Maryland bipolar forceps are less optimal for blunt dissection than monopolar scissors. Consequently, DBM may not be the optimal choice for procedures requiring extensive blunt dissection, such as radical nephrectomy and nephroureterectomy.

Robot-assisted surgery is rapidly spreading in all surgical specialties in Japan. Many surgical procedures traditionally performed by laparotomy or laparoscopy are increasingly being performed using robotics; thus, opportunities for robotic repair of genitourinary injuries are also increasing [11,12]. It is expected that opportunities for combined surgery with other specialties will increase, necessitating a certain level of familiarity with procedures and equipment used in other specialties.

## Conclusions

Robotic-assisted partial cystectomy using the DBM was technically feasible. When performing combined procedures with other departments, if the DBM is already being used, it is worthwhile to attempt to reduce surgical costs.
